# Editorial: Immunometabolism of T cells in skin infection, autoimmunity and cancer biology

**DOI:** 10.3389/fimmu.2023.1237386

**Published:** 2023-06-30

**Authors:** Lu Peng, Ling Chen, Laura A. Solt, Venina Marcela Dominical, Zhu Shen

**Affiliations:** ^1^ Department of Dermatology, Guangdong Provincial People’s Hospital (Guangdong Academy of Medical Sciences), Southern Medical University, Guangzhou, China; ^2^ Department of Dermatology, Daping Hospital, Army Medical University, Chongqing, China; ^3^ Department of Immunology and Microbiology, The Herbert Wertheim UF Scripps Institute for Biomedical Innovation & Technology, The Scripps Research Institute, Jupiter, FL, United States; ^4^ Department of Research and Development, Santa Ana Bio, Inc., Alameda, CA, United States

**Keywords:** immunometabolism, T cells, regulatory T cells, memory T cells, autoimmunity, tumor, extracellular environment

Subsets of immune cells exhibit distinct functional capacities and utilize different metabolic pathways to orchestrate their differentiation, activation, function, plasticity, and survival (1). Understanding how metabolic reprogramming instructs T-cell subsets not only represents crucial opportunities to delineate new avenues in their biology, but also provides opportunities for tuning T-cell function for therapeutic purposes (2). In this Research Topic, the authors have reviewed current investigations and understanding of the immunometabolism of T-cell subsets and elucidated a series of metabolic reprogramming strategies for enhancing antitumor function, improving transplantation immunity, and ameliorating autoimmune diseases.

Several obstacles, including inadequate immune infiltrate, a suppressive tumor microenvironment (TME), tumor heterogeneity and evolution, and exhausted effector cells, pose significant challenges to the sustained antitumor ability of genetically engineered T cells in promising T cell receptor targeting (TCR-T) therapies for solid tumors (3, 4). To overcome these obstacles, Mao provided a multidimensional review of current metabolic reprogramming strategies. These strategies include pharmacologic inhibition of glucose-6-phosphate dehydrogenase (G6PD) and glutamine transporters to enhance T cell cytotoxicity, treatment with Diclofenac to inhibit monocarboxylate transporter 1 and 4 (MCT1 and MCT4), indoleamine 2,3-dioxygenase (IDO) inhibitors such as Epacadostat to reduce kynurenine (5, 6). As metabolism is a matter of balance, perturbations must be timed precisely to yield the desired phenotype. Mao emphasized that, due to the complexity of targeting mTORC1, targeting mTORC2 could emerge as a potential alternative to enhance potent and long-lived T cell responses (6). Furthermore, systemic inhibition of acetyl-CoA carboxylase would need to be carefully timed to avoid restricting T cell expansion (7). Similarly, use of AMPK agonists should be approached with care to improve immune cell survival while simultaneously reducing the flux through the glycolytic pathway in cancer cells (8).

Analysis of immunotherapy outcomes indicates that stem-like or memory T cell populations correlate with superior responses to cancer (9, 10). In their Mini-Review, Claiborne highlighted the therapeutic implications of metabolic factors for maintaining memory phenotypes within the TME, such as culturing engineered T cells with IL-7 or IL-15, or inhibiting lactate, adenosine, and canine uric acid. Pharmacological intervention strategies were also mentioned to overcome the inhibitory signals of the TME, such as high concentrations of extracellular potassium ions, tissue hypoxia, ROS accumulation, and decreased mitochondrial quality. Furthermore, the Mini-Review summarized epigenetic regulation *via* vitamin A metabolism and also the newly discovered anticancer effect of vitamin E (11, 12). These master regulators of energy metabolism and epigenetic regulation of critical loci have been manipulated in pre-clinical settings, and balancing multiple interventions may be the future of the field.

CD4+CD25^high^FoxP3+ regulatory T cells (Tregs) are important targets of immunometabolism regulation due to their crucial immunosuppressive functions and special metabolic preferences. Bulygin et al. and Tomaszewicz et al. reviewed metabolic reprogramming strategies for Tregs, focusing on tumor and transplantation immunity and autoimmune diseases, respectively. Bulygin et al. first described plasticity and tissue-specific phenotypes in Treg development under specific environmental signals. They also pointed out potential metabolic reprogramming targets by manipulating TCR/CD28 signaling and the PI3K-AKT-mTORC1 cascade, which are critical for FOXP3 expression and Treg formation (13, 14). The authors further highlighted the pivotal role of glycolysis and fatty acid oxidation (FAO) in determining Treg differentiation and function during tumor growth and after transplantation. Tomaszewicz et al. described the distinct metabolic and transcriptional characteristics of Tregs in different states (resting and activated) and sites of residence (central and peripheral). They detailed the endogenous metabolic factors affecting Treg stability, such as the balance between glucose and lipid metabolism and the mTOR pathway, as well as external factors like ischemic environments, inflammation, and adenosine. Additionally, they emphasized low dose IL-2 treatment contributed to the proliferation and maintenance of Tregs (15). The authors also elaborated on how drugs commonly used for autoimmune diseases may affect the metabolism of immune cells. However, as discussed by Bulygin et al., several questions remain, such as how to balance metabolism and immunological function of Tregs under constantly varying environmental conditions, and how to fully switch Treg metabolism from “classic” aerobic glycolysis to an alternative energy source in the form of oxidative phosphorylation *via* FAO.

Immune cells can also exhibit heterogeneity in different anatomical sites and histological structures, and it may be related to differences in nutrient metabolism. Sato et al. and Lyu and Sun provided their contributions to exploring immune reprogramming in the field of dermatology. Sato et al. delineated an in-depth phenotype and function of resident memory Tregs in the human epidermis, which differs from previous studies occurring in full-layer skin. They showed that Foxp3 was highly demethylated and selectively expressed in human epithelial CD4+CD103-T cells. Epidermal CD4+CD103-T cells have higher IL-2Rα (CD25) and IL-2Rβ (CD122) expressions and are equipped with a greater capability of suppressing proliferation of other resident memory T cells compared with CD4+CD103+T cells. These results suggest that the CD103- population could be the focus of Treg immunometabolism intervention in epidermal and mucosal regions, and they may be more susceptible to Foxp3 interference and IL-2 inhibitors.

Studies have revealed that vitiligo, especially the most common nonsegmental type, is closely associated with systemic metabolic disorders. Oxidative stress, abnormal glucose and lipid metabolism, ferroptosis and T-cell dysfunction in vitiligo were observed (16). Lyu and Sun reviewed the biological mediators and molecular mechanisms underlying metabolic defects and detailed potential therapeutic targets of metabolic reprogramming based on recent research. For instance, the JAK-STAT pathway is pivotal for cellular metabolism (17). Interfering with lipid metabolism and applying antibodies against CD122 are promising approaches for inhibiting the function and survival of tissue-resident memory T cells that mediate vitiligo reactivation (18).

Taken together, immune cell immunometabolism is dynamic, occurring during cellular differentiation and functional stages, enabling them to adapt to the ever-changing extracellular environment. The applications based on these investigations have great clinical potential, but further exploration is needed to see them enter the clinic.

## Author contributions

LP performed literature research and wrote the manuscript. LC, LS, VD and ZS performed literature research. ZS, LC, LS and VD revised the manuscript. All authors contributed to the article and approved the submitted version.
